# Distinct functions and regulation of epithelial progesterone receptor in the mouse cervix, vagina, and uterus

**DOI:** 10.18632/oncotarget.8159

**Published:** 2016-03-17

**Authors:** Fabiola F. Mehta, Jieun Son, Sylvia C. Hewitt, Eunjung Jang, John P. Lydon, Kenneth S. Korach, Sang-Hyuk Chung

**Affiliations:** ^1^ Center for Nuclear Receptors and Cell Signaling, Department of Biology and Biochemistry, University of Houston, Houston, TX, USA; ^2^ Reproductive and Developmental Biology Laboratory, National Institute of Environmental Health Sciences, Research Triangle Park, NC, USA; ^3^ Department of Molecular and Cellular Biology, Baylor College of Medicine, Houston, TX, USA

**Keywords:** progesterone receptor, estrogen receptor α, epithelium, female reproductive tract, mouse model, Pathology Section

## Abstract

While the function of progesterone receptor (PR) has been studied in the mouse vagina and uterus, its regulation and function in the cervix has not been described. We selectively deleted epithelial PR in the female reproductive tracts using the *Cre/LoxP* recombination system. We found that epithelial PR was required for induction of apoptosis and suppression of cell proliferation by progesterone (P_4_) in the cervical and vaginal epithelium. We also found that epithelial PR was dispensable for P_4_ to suppress apoptosis and proliferation in the uterine epithelium. PR is encoded by the *Pgr* gene, which is regulated by estrogen receptor α (ERα) in the female reproductive tracts. Using knock−in mouse models expressing ERα mutants, we determined that the DNA−binding domain (DBD) and AF2 domain of ERα were required for upregulation of *Pgr* in the cervix and vagina as well as the uterine stroma. The ERα AF1 domain was required for upregulation of *Pgr* in the vaginal stroma and epithelium and cervical epithelium, but not in the uterine and cervical stroma. ERα DBD, AF1, and AF2 were required for suppression of *Pgr* in the uterine epithelium, which was mediated by stromal ERα. Epithelial ERα was responsible for upregulation of epithelial *Pgr* in the cervix and vagina. Our results indicate that regulation and functions of epithelial PR are different in the cervix, vagina, and uterus.

## INTRODUCTION

Progesterone (P_4_) and estradiol (E_2_) are major ovarian steroid hormones crucial for the development and homeostasis of the female reproductive tract (i.e., uterus, cervix, and vagina) [1]. P_4_ and E_2_ function through progesterone receptor (PR) and estrogen receptor (ER), respectively. PR and ER are ligand−dependent transcription factors belonging to the nuclear receptor superfamily [1]. ERα and ERβ are encoded by *Esr1* and *Esr2,* respectively [2]. ERα is the major ER subtype in the female reproductive tract of mice and humans [2–4]. ERα is composed of several distinct functional domains, N−terminal activation function 1 (AF1), DNA−binding domain (DBD), a hinge region, and C−terminal ligand−binding domain containing the AF2. ERα activates its target gene expression by binding to estrogen response elements (EREs) through its DBD (classical mechanism) or other transcription factor (e.g., AP-1 and Sp-1) binding sites through protein−protein interactions (tethering mechanism) [2]. The *Pgr* gene coding for PR is a well−known ERα target gene in the female reproductive tract [1, 5]; however, the mechanism of *Pgr* activation by ERα is poorly understood. Reporter assays using isolated regulatory sequences of the *PGR* gene have implicated both classical and tethering mechanism in the transcriptional activation of *PGR* in MCF7 breast cancer cells [6–9]. However, it is unclear whether it is relevant to the normal female reproductive tract and whether both pathways are important in the natural chromatin context.

During the female sexual cycle in humans and rodents, an E_2_ surge promotes and a P_4_ surge inhibits epithelial cell proliferation in the reproductive tracts [10]. The uterine and vaginal epithelia consist of columnar and squamous epithelial cells, respectively. The cervical epithelium, however, is composed of columnar and stratified squamous epithelial cells [11]. The hormone−mediated regulation of epithelial cell proliferation in the reproductive tracts involves crosstalk between the stromal and epithelial compartment [12]. Epithelial ERα is dispensable for E_2_−induced proliferation of uterine columnar epithelial cells [13]. Stromal ERα is required for squamous epithelial cell proliferation in the cervix and vagina [14]. Epithelial PR is dispensable for P_4_−induced suppression of epithelial cell proliferation in the uterine tissue recombinants derived from neonatal mice [15]. P_4_ suppresses apoptosis in the uterine epithelium, which is mediated by stromal PR [16]. While PR is expressed in the cervical stroma and epithelium [17], knowledge on the function of PR and effects of P_4_ in the cervix is limited.

Using knock−in mice expressing mutant ERα lacking activities of DBD, AF1, or AF2, we show that all three domains are required for upregulation of *Pgr* in the cervical epithelium and vagina. We also show that AF1 is dispensable for upregulation of *Pgr* in the cervical and uterine stroma. Unlike the uterus, P_4_ promotes epithelial apoptosis in the cervix and vagina. P_4_ inhibits E_2_−induced cervical and vaginal squamous cell proliferation. Using epithelial *Pgr*−deficient mice, we demonstrate that epithelial PR is required for P_4_−induced apoptosis and suppression of cell proliferation in the cervical and vaginal epithelium. Our results are the first to show different functions and regulation of epithelial PR in the cervix, vagina, and uterus under the same hormonal condition.

## RESULTS

### Histological features of the female reproductive tract lacking epithelial PR

**Figure 1:**
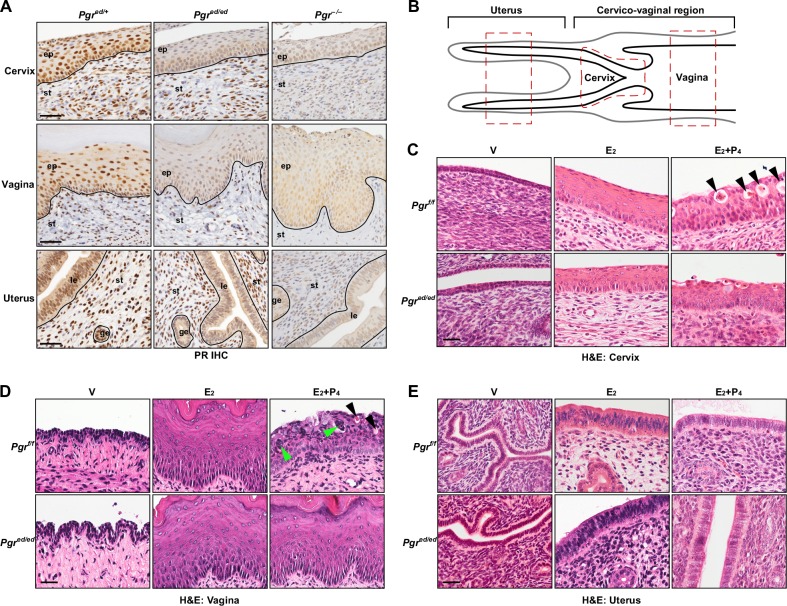
Histology of female reproductive tracts in epithelial PR−deficient mice treated with E_2_+P_4_ **A.** Epithelium−specific deletion of PR. Serial sections of the cervix, vagina, and uterus were stained for PR (brown). Nuclei were counterstained with hematoxylin. Black lines separate cervical/vaginal squamous epithelium (ep) and uterine luminal (le)/glandular epithelium (ge) from stroma (st). Scale bar, 50 μm. **B.** Schematics of murine female reproductive tracts. All analyses were carried out with red−boxed areas of each tissue. Note that a region between the cervix and uterus is not annotated because there is no histological boundary. **C.**-**E.** Ovariectomized mice were treated with female sex hormones as indicated and paraffin sections of the cervix **C.**, vagina **D.**, and uterus **E.** were subjected to H&E staining. Black arrowheads in **C.** and **D.** indicate cells undergoing hydropic degeneration. Green arrowheads in **D.** indicate non−epithelial cells. Representative images of each group are shown (*n* = 3-5). V, vehicle; E_2_, estradiol; P_4_, progesterone, Scale bar, 30 μm.

To interrogate a role of epithelial PR in responses of the female reproductive tract to P_4_, we made use of *Wnt7a−Cre/Pgr^f/f^* mice (referred to as *Pgr^ed/ed^* hereafter; ed, epithelial deletion). In these mice, PR expression was ablated in the epithelium, but not stroma, of the cervix, vagina, and uterus (Figure 1A). To characterize epithelial PR functions under the same hormonal condition, we treated ovariectomized mice with E_2_ and P_4_ for 7 days. Seven−day treatment with E_2_ was required for the entire cervical epithelia to reach the full thickness (Supplementary Figure S1). Regions of the uterus, cervix, and vagina that were analyzed throughout the study are shown in Figure 1B. The cervical epithelia of vehicle−treated *Pgr^f/f^* and *Pgr^ed/ed^* mice were thin (13.2 μm in average) and composed of 2−3 cell layers (Figure 1C). E_2_ increased the height of the cervical epithelium in *Pgr^f/f^* (65.8 ± 4.2 μm) and *Pgr^ed/ed^* mice (66.6 ± 2.9 μm) (Figure 1C). The cervical epithelium height in E_2_+P_4_−treated *Pgr^f/f^* (57.5 ± 5.0 μm) and *Pgr^ed/ed^* mice (60.7 ± 4.6 μm) was not significantly different from genotype−matching mice treated with E_2_ alone. The nucleus/cytoplasm ratio (0.66 ± 0.03) of cervical suprabasal cells in E_2_−treated *Pgr^f/f^* mice was significantly smaller than the ratio (1.09 ± 0.06) in E_2_+P_4_−treated mice (*P* = 0.05). A similar difference was observed in *Pgr^ed/ed^* mice [0.66 ± 0.02 (E_2_) *vs*. 1.03 ± 0.01 (E_2_+P_4_); *P* = 0.05]. Hydropic degeneration was prominent in the cervix of E_2_+P_4_−treated *Pgr^f/f^* but not *Pgr^ed/ed^* mice (Figure 1C). E_2_ induced hyperplasia and keratinization in the vaginal epithelium of *Pgr^f/f^* and *Pgr^ed/ed^* mice (Figure 1D). In the vaginal epithelium of E_2_+P_4_−treated *Pgr^f/f^* mice, keratinization was absent and hydropic degeneration was observed (Figure 1D). Also found was infiltration of K14−negative non−epithelial cells (Supplementary Figure S2), which are likely neutrophils [18]. These P_4_−induced histologic changes in the vaginal epithelium were absent in E_2_+P_4_−treated *Pgr^ed/ed^* mice (Figure 1D). E_2_ induced hyperplasia in the uterine epithelium, which was reversed by P_4_ in both *Pgr^f/f^* and *Pgr^ed/ed^* mice (Figure 1E). Phenotypes described in this study are summarized in Table 1.

**Table 1:** Summary of P_4_−induced phenotypes in the female reproductive tract^1^

	Apoptosis				Proliferation				N/C ratio^2^				K10 expression				Alcian blue staining & hydropic degeneration				Keratinization			
	Cervix/Vagina		Uterus		Cervix/Vagina		Uterus		Cervix		Vagina		Cervix		Vagina		Cervix		Vagina		Cervix		Vagina	
Treat.	E_2_	E_2_+P_4_	E_2_	E_2_+P_4_	E_2_	E_2_+P_4_	E_2_	E_2_+P_4_	E_2_	E_2_+P_4_	E_2_	E_2_+P_4_	E_2_	E_2_+P_4_	E_2_	E_2_+P_4_	E_2_	E_2_+P_4_	E_2_	E_2_+P_4_	E_2_	E_2_+P_4_	E_2_	E_2_+P_4_
*Pgr^f/f^*	+	++	++	+	++	+	++	+	+	++	+	++	++	+	++	+	−	++	−	+	−	−	++	−
*Pgr^ed/ed^*	+	+	++	+	++	++	++	+	+	++	+	+	++	+	++	++	−	−	−	−	−	−	++	++

1 Shown are phenotypes in epithelial PR–sufficient (*Pgrf/f*) and –deficient (*Pgred/ed*) mice treated with E_2_ and E_2_+P_4_. 2Nucleus to cytoplasm ratio. ++, high; +, low; −, rare/absent.

### P_4_ fails to inhibit epithelial cell proliferation in the cervix and vagina, but not uterus of *Pgr^ed/ed^* mice

**Figure 2:**
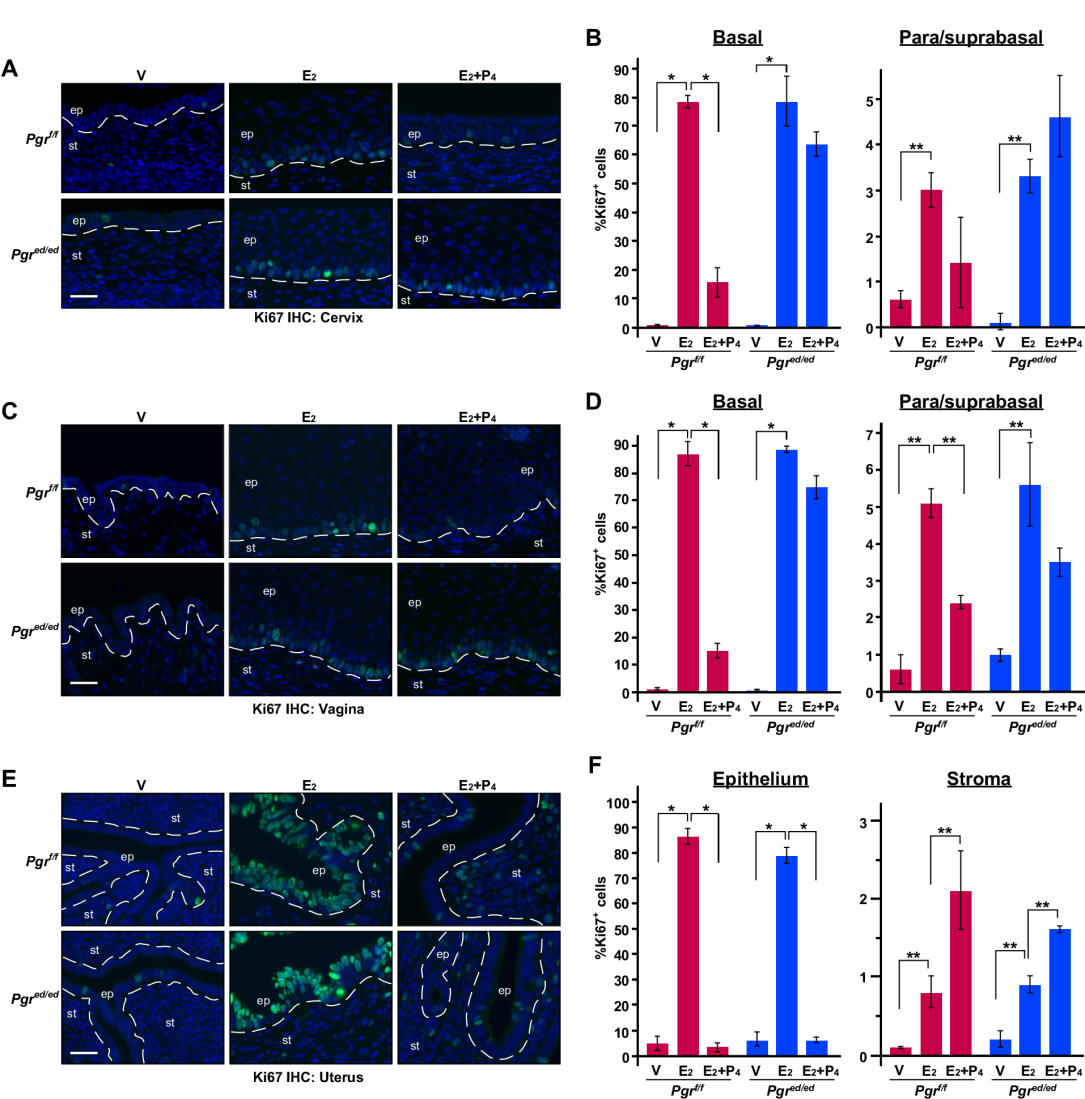
Epithelial PR is required for P_4_ to inhibit epithelial cell proliferation in the cervix and vagina, but not uterus Ovariectomized mice were treated with female sex hormones as indicated. Paraffin sections of the cervix **A.**, vagina **C.**, and uterus **E.** were stained for Ki67 (green). Nuclei were stained with Hoechst 33342 (blue). Scale bars, 30 μm. Results were quantified as described in *Materials & Methods*. Quantified results of the cervix **B.**, vagina **D.**, and uterus **F.** are shown as mean ± S.E.M (*n* = 3-5 per group). *P ≤ 0.01, **P ≤ 0.05.

P_4_ inhibits epithelial cell proliferation in the female reproductive tract [15, 19]. We sought to determine whether epithelial PR is required for this effect. We analyzed expression of cell proliferation marker Ki67 to measure cell proliferation. Less than 1% of cervical epithelial cells were proliferative in vehicle−treated *Pgr^f/f^* and *Pgr^ed/ed^* mice (Figure 2A-2B). E_2_−induced cervical epithelial cell proliferation was not different between the two genotypes. Compared to genotype−matching mice treated with E_2_ alone, cervical epithelial cell proliferation in the basal layer was significantly decreased in E_2_+P_4_−treated *Pgr^f/f^* but not *Pgr^ed/ed^* mice (Figure 2A-2B). Similar phenotypes were observed in the vaginal epithelium of *Pgr^f/f^* and *Pgr^ed/ed^* mice (Figure 2C-2D and Table 1). In the uteri of both *Pgr^f/f^* and *Pgr^ed/ed^* mice, E_2_−induced epithelial cell proliferation was significantly inhibited by P_4_ (Figure 2E-2F). In both genotypes, however, stromal cell proliferation was increased in the uteri of E_2_+P_4_−treated mice compared to E_2_−treated mice (Figure 2F). Similar results were obtained in the columnar epithelium of the cervix (data not shown). These results indicate that, under the same hormonal condition, epithelial PR is required for P_4_−induced suppression of cell proliferation only in the cervical and vaginal squamous epithelium.

### Epithelial PR is required for proper differentiation of cervical and vaginal squamous epithelial cells

**Figure 3:**
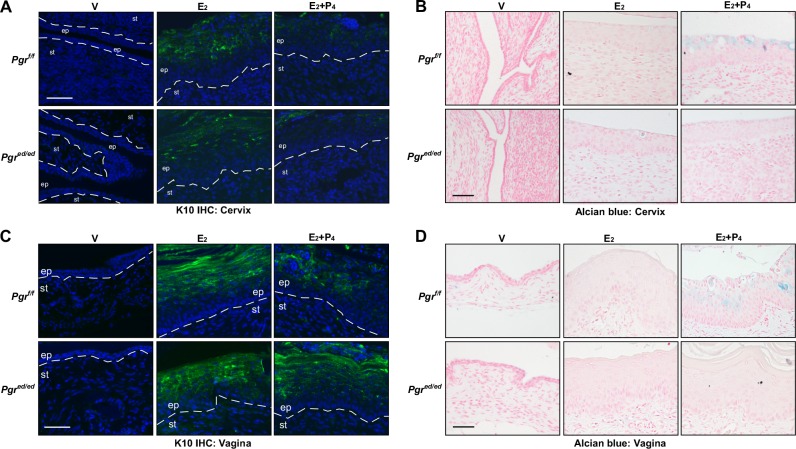
Epithelial PR is required for P_4_−mediated regulation of differentiation in the cervical and vaginal epithelium Reproductive tracts were isolated from female mice that were ovariectomized and then treated as indicated. Representative images of each group are shown (*n* = 3). **A.** Epithelial PR is dispensable for suppression of K10 expression by P_4_ in the cervical epithelium. Cervical sections were stained for K10 (green) and nuclei were stained with Hoechst 33342 (blue). Dotted lines separate epithelium (ep) and stroma (st). Scale bar, 50 μm. **B.** Alcian blue staining is increased by P_4_ in an epithelial PR−dependent manner in the cervical epithelium. Cervical sections were stained with Alcian blue. Nuclei were counterstained with Nuclear Fast Red. Scale bar, 50 μm. **C.** Epithelial PR is necessary for suppression of K10 expression by P_4_ in the vaginal epithelium. Vaginal sections were stained for K10 (green) and nuclei were stained with Hoechst 33342 (blue). Scale bar, 50 μm. **D.** Alcian blue staining is increased by P_4_ in an epithelial PR−dependent manner in the vaginal epithelium. Vaginal sections were subjected to Alcian blue staining. Nuclei were counterstained with Nuclear Fast Red. Scale bar, 50 μm.

Histological features observed in the cervical and vaginal epithelium (*see* Figure 1C-1D) suggested that P_4_ regulates differentiation of squamous cells. Thus, we carried out Alcian blue staining and immunohistochemistry for cytokeratin 10 (K10), squamous differentiation marker [20]. Alcian blue staining is typically used to detect cervical acidic mucins, which are expressed by differentiated cells [21]. As expected, E_2_ induced expression of K10 in the suprabasal layer in the cervical epithelia of *Pgr^f/f^* and *Pgr^ed/ed^* mice (Figure 3A). K10 expression was reduced in the cervical epithelia of E_2_+P_4_−treated *Pgr^f/f^* and *Pgr^ed/ed^* mice, indicating that epithelial PR is dispensable for K10 suppression by P_4_. Alcian blue staining was prominent in the cervix of *Pgr^f/f^* mice treated with E_2_+P_4_, but absent in identically treated *Pgr^ed/ed^* mice (Figure 3B). This result indicates that P_4_−promoted Alcian blue staining is dependent upon epithelial PR. P_4_ inhibited E_2_−induced K10 expression and increased Alcian blue staining in the vaginal epithelium of *Pgr^f/f^* but not *Pgr^ed/ed^* mice (Figure 3C-3D and Table 1). These results indicate that epithelial PR is required for P_4_−mediated regulation of squamous cell differentiation in the vagina to the greater extent than in the cervix.

### Epithelial PR plays distinct roles in epithelial apoptosis in the lower and upper reproductive tracts

**Figure 4:**
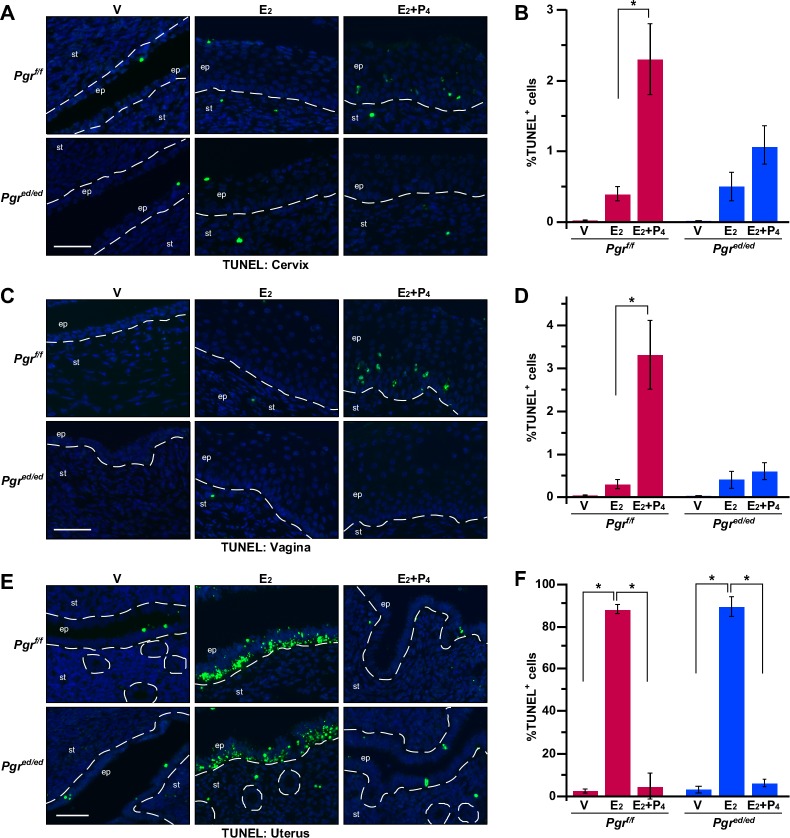
Epithelial PR is required for P_4_−mediated regulation of apoptosis in the cervix and vagina, but not the uterus Reproductive tracts were isolated from female mice that were ovariectomized and then treated as indicated. **A.** P_4_ induces apoptosis in the cervical epithelium through epithelial PR. Cervical sections were subjected to TUNEL staining (green). Nuclei were stained with Hoechst 33342 (blue). Dotted lines separate epithelium (ep) and stroma (st). Scale bar, 50 μm. **B.** Results shown in **A.** were quantified. Results are shown as mean ± S.E.M (*n* = 3-5 per group). *P = 0.04. **C.** P_4_ induces apoptosis in the vaginal epithelium through epithelial PR. Vaginal sections were subjected to TUNEL staining (green). Scale bar, 50 μm. **D.** Results shown in **C.** were quantified and displayed as mean ± S.E.M (*n* = 3-5 per group). *P = 0.04. **E.** P_4_ prevents apoptosis in an epithelial PR−independent manner in the uterine epithelium. Uterine sections were subjected to TUNEL staining (green). Scale bar, 50 μm. **F.** Results shown in **E.** were quantified and displayed as mean ± S.E.M (*n* = 3 per group). *P = 0.05.

P_4_ inhibits apoptosis in the uterine epithelium [16]. We carried out TUNEL assays to determine the role of P_4_ and epithelial PR in regulation of apoptosis in the cervix, vagina, and uterus. A few TUNEL−positive apoptotic cells were found in the cervical epithelium of vehicle− and E_2_−treated *Pgr^f/f^* and *Pgr^ed/ed^* mice (Figure 4A-4B). Compared to E_2_−treated mice, the apoptotic index was increased in the cervical epithelium of E_2_+P_4_−treated *Pgr^f/f^* but not *Pgr^ed/ed^* mice (Figure 4A-4B). Similar results were obtained in the vaginal epithelium (Figure 4C-4D and Table 1). E_2_ induced apoptosis in the uterine epithelium regardless of the genotype, which was inhibited by P_4_ in both *Pgr^f/f^* and *Pgr^ed/ed^* mice (Figure 4E-4F and Table 1). These results indicate that epithelial PR is required for P_4_−induced apoptosis in the epithelium of lower reproductive tracts (i.e., cervix and vagina), but dispensable for P_4_ to inhibit apoptosis in the uterine epithelium.

### ERα DBD and AF2 are required for upregulation of *Pgr* in the cervix and vagina

**Figure 5:**
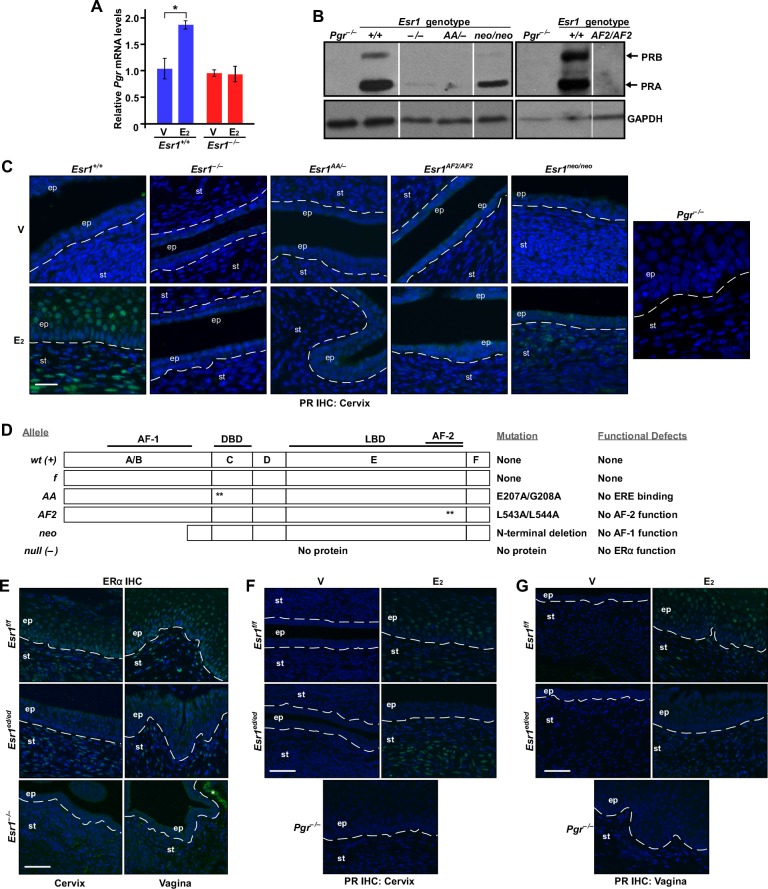
Mechanisms of upregulation of *Pgr* by ERα in the cervix and vagina **A.** *Pgr* is upregulated by E_2_−ERα in the cervix. Mice were ovariectomized and treated with E_2_ for 24 hr. The relative levels of *Pgr* mRNA were compared by qPCR. The value from vehicle−treated *Esr1*^+/+^ cervix was set at 1. Results are shown as mean ± S.E.M (*n* = 3). *P = 0.05. **B.** Upregulation of PR expression is absent in the cervix of *Esr1*^AA/−^ and *Esr1*^AF2/AF2^ mice. Ovary−intact mice of indicated genotypes (8-12 weeks of age) were treated with E_2_ for 3 days to synchronize mice at the estrus. Expression of PR in the cervix was determined by immunoblot. *Pgr*^−/−^ tissue extracts were used as negative control. GAPDH was used as loading control. Results were taken from separate gels indicated by black boxes. Intervening lanes in a same gel were removed, which was indicated by vertical white lines. Representative results from more than 2 mice per genotype are shown. **C.** E_2_−induced PR expression is absent in the cervix of *Esr1*^AA/−^ and *Esr1*^AF2/AF2^ mice. Ovariectomized mice (*n* = 3 per group) were treated with vehicle or E_2_ for 7 days. Cervical tissue sections were stained for PR (green). Nuclei were stained with Hoechst 33342 (blue). Dotted lines separate epithelium (ep) and stroma (st). Scale bar, 25 μm. **D.** Summary of ERα proteins expressed by *Esr1* alleles used in the study. Asterisks denote locations of alanine substitution mutations. AF, activation function; DBD, DNA−binding domain; LBD, ligand−binding domain. **E.** *Esr1* is deleted in the cervical and vaginal epithelium. Cervical and vaginal sections (*n*= 3 per tissue) were stained for ERα (green). Nuclei were stained with Hoechst 33342 (blue). Scale bar, 50 μm. **F.**-**G.** Epithelial ERα is required for upregulation of epithelial *Pgr* in the lower reproductive tracts. Mice (*n* = 3 per group) were ovariectomized and treated with vehicle or E_2_. Tissue sections of the cervix **F.** and vagina **G.** were stained for PR (green). Nuclei were stained with Hoechst 33342 (blue). Scale bar, 50 μm.

ERα regulates transcription of *Pgr* in the vagina and uterus [5]. We found that E_2_ increased the *Pgr* mRNA level significantly in the cervix of *Esr1^+/+^* but not *Esr1^−/−^* mice (Figure 5A). Similarly, PR protein was readily detected in cervical tissue extracts and paraffin sections derived from *Esr1^+/+^* but not *Esr1^−/−^* mice (Figure 5B-5C). These results demonstrate that ERα activates transcription of *Pgr* in the cervix. To understand a mechanism of transcriptional activation of *Pgr* by ERα, we made use of knock−in mouse models expressing ERα mutant defective for DBD, AF1, or AF2 (Figure 5D). The *AA* allele expresses an ERα mutant (E207A/G208A) that lacks the ERE−binding activity [22]. The *AF2* allele expresses ERα harboring L543A/L544A substitution mutations in the helix 12, which abrogates the AF2 function [23]. The *neo* allele has a neo cassette in the exon 2, which is designed to abrogate expression of ERα [24]. This allele, however, expresses truncated ERα proteins lacking the AF1 domain due to cryptic splicing events [25, 26]. PR expression was barely detectable in cervical tissue extracts obtained from *Esr1^AA/−^* and *Esr1^AF2/AF2^* mice (Figure 5B). Similarly, E_2_ failed to induce PR expression in the cervix of *Esr1^AA/−^* and *Esr1^AF2/AF2^* mice (Figure 5C). Albeit reduced compared to *Esr1^+/+^* mice, the PR level was increased in cervical tissue extracts from *Esr1^neo/neo^* mice compared to *Esr1^−/−^*, *Esr1^AA/−^*, and *Esr1^AF2/AF2^* mice (Figure 5B). Upregulation of PR expression in the cervix of *Esr1^neo/neo^* mice was mostly restricted in the stroma (Figure 5C). E_2_ did not activate *Pgr* expression in the vagina of *Esr1^−/−^*, *Esr1^AA/−^*, *Esr1^AF2/AF2^*, and *Esr1^neo/neo^* mice (Supplementary Figure S3). PR expression patterns were similar in the cervix and vagina of *Esr2^+/+^* and *Esr2^−/−^* mice (Supplementary Figure S4), indicating that ERβ is not required for upregulation of *Pgr*. To determine whether stroma−epithelium cross talk is involved in upregulation of *Pgr* by E_2_−ERα, we used *Wnt7a−Cre/Esr1^f/f^* (referred to as *Esr1^ed/ed^* hereafter). ERα expression was efficiently ablated in epithelial but not stromal cells in the cervix and vagina of *Esr1^ed/ed^* mice (Figure 5E). While E_2_ induced PR expression in the cervical stroma of *Esr1^f/f^* and *Esr1^ed/ed^* mice, E_2_ failed to do so in the cervical epithelium of *Esr1^ed/ed^* mice (Figure 5F). Similar results were obtained from the vagina (Figure 5G). These results suggest that, in the cervix and vagina, E_2_−induced upregulation of *Pgr* is cell−autonomous and mediated commonly by ERα DBD (i.e., ERE−binding) and AF2.

### ERα DBD and AF2 are required for upregulation of stromal *Pgr* and downregulation of epithelial *Pgr* in the uterus

**Figure 6:**
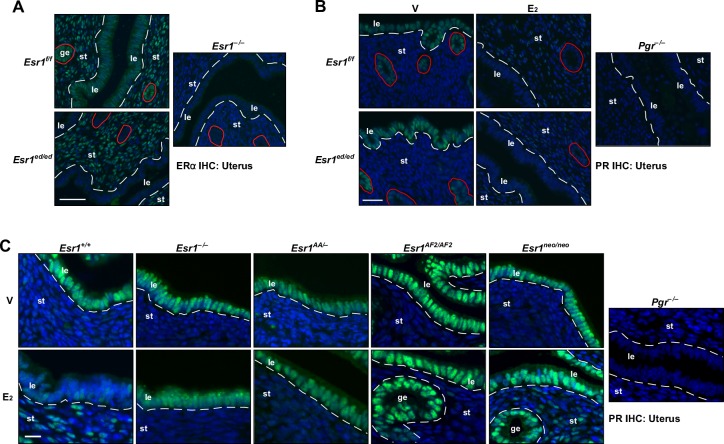
Mechanisms of regulation of *Pgr* by ERα in the uterus **A.** *Esr1* is deleted in the uterine epithelium. Uterine sections (*n* = 3) were stained for ERα (green). Nuclei were stained with Hoechst 33342 (blue). Dotted lines separate luminal epithelium (le) and stroma (st). Glandular epithelia (ge) are circumscribed by red circles. Scale bar, 50 μm. **B.** E_2_ suppresses epithelial *Pgr* expression in the *Esr1*^ed/ed^ uteri. Mice (*n* = 3) were ovariectomized and treated with vehicle or E_2_. Uterine tissue sections were stained for PR (green). Nuclei were stained with Hoechst 33342 (blue). Dotted lines separate luminal epithelium (le) and stroma (st). Glandular epithelia (ge) are circumscribed by red circles. Note that E_2_ downregulates epithelial *Pgr* and upregulates stromal *Pgr* in both genotypes. Scale bar, 25 μm. **C.** ERα DBD and AF2 are required for *Pgr* regulation in the uterus. Mice were treated as described in **B.**. Uterine tissue sections (*n* = 3 per group) were stained for PR (green). Nuclei were stained with Hoechst 33342 (blue). Dotted lines are to separate luminal (le) and glandular epithelium (ge) from stroma (st). Scale bar, 30 μm.

Consistent with previously published results [13], expression of ERα was lost in the epithelium but retained in the stroma of the *Esr1^ed/ed^* uteri (Figure 6A). While stromal PR expression was increased by E_2_ in the *Esr1^f/f^* and *Esr1^ed/ed^* uteri, E_2_ decreased the PR levels in the uterine epithelium in both genotypes (Figure 6B). This result confirmed the paracrine mechanism of *Pgr* downregulation by ERα in the uterine epithelium [5, 27]. Stromal upregulation and epithelial downregulation of *Pgr* by E_2_ were absent in the uterus of *Esr1^−/−^*, *Esr1^AA/−^*, and *Esr1^AF2/AF2^* mice (Figure 6C). As reported previously [5], E_2_ activated stromal *Pgr* expression but failed to downregulate epithelial *Pgr* in the uterus of *Esr1^neo/neo^* mice (Figure 6C). In the *Esr2^−/−^* uterus, E_2_ increased and decreased stromal and epithelial PR expression, respectively (Supplementary Figure S4), indicating that ERβ is not required for regulation of *Pgr* in the uterus. These results indicate that the function of ERα DBD and AF2 in stromal cells is required for stromal upregulation and epithelial downregulation of *Pgr* in the uterus.

**Figure 7:**
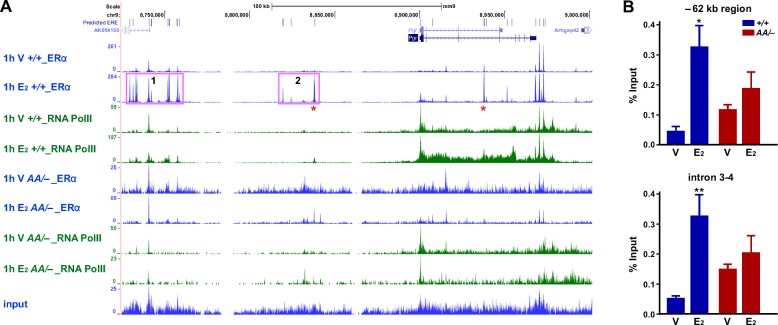
ERα binding to EREs correlates with RNA polII recruitment to the *Pgr* locus in the uterus **A.** E_2_ induces binding of ERα and RNA polII to *Pgr* in the uterus of *Esr1*^+/+^ but not *Esr1*^AA/−^ mice. Recruitment of ERα and RNA polII to the *Pgr* locus was analyzed using ChIP−seq data available in Gene Expression Omnibus (*Esr1*^+/+^, GSE36455l; *Esr1*^AA/−^, GSE56466). Vertical blue lines at the top indicate locations of predicted EREs, which were identified using position weight matrix algorithm. Two peaks validated in **B.** are indicated by red asterisk. Pink boxes indicate −155 kb (1) and −72 kb cluster (2). **B.** Validation of E_2_−dependent enrichment of ERα at two candidate binding regions. Mice were ovariectomized and treated with vehicle or E_2_. Extracted chromatin was subjected to ERα ChIP and enrichment of approximately 62 kb upstream of the *Pgr* TSS and intron 3-4 were quantified by qPCR. Data normalized to % input DNA is shown as mean ± S.E.M. (*n* = 4-5). *P = 0.001; **P = 0.0005.

We next analyzed binding of ERα to the *Pgr* gene using the previously published ERα ChIP−seq data [28, 29]. Approximately 200 kb upstream and 100 kb downstream of the *Pgr* transcription start site (TSS) were analyzed for the binding of wild−type ERα. While there were two E_2_−independent ERα−binding peaks at the 3′ end of the *Pgr* gene, E_2_ enhanced ERα binding to the intragenic regions and −72 kb and −155 kb cluster in the *Esr1^+/+^* uterus (Figure 7A). E_2_ also increased RNA polII binding to the *Pgr* gene in the same tissue. While the −155 kb cluster was closer to *AK054106*, E_2_ did not regulate this gene (data not shown). Notably, we identified predicted half or full EREs in all ERα−binding sites (Figure 7A). Consistently, in the uterus of *Esr1^AA/−^* mice, both E_2_−dependent and −independent ERα binding to the *Pgr* locus were not observed (Figure 7A). Concordantly, E_2_−induced recruitment of RNA polII was absent in the *Esr1^AA/−^* uterus. We confirmed by ChIP followed by qPCR that E_2_ induced enrichment of ERα binding to the −62 kb region (7.1-fold) and intron 3-4 (6.2-fold) of *Pgr* in the uterus of *Esr1^+/+^* but not *Esr1^AA/−^* mice (Figure 7B). These results suggest that, in the uterus, ERα activates transcription of *Pgr* mainly by binding to EREs.

## DISCUSSION

P_4_ inhibits E_2_−promoted epithelial cell proliferation in the murine female reproductive tract [19, 30]. We showed that, under the same hormonal condition, epithelial PR was necessary for P_4_ to suppress E_2_−induced epithelial cell proliferation in the cervix and vagina, but dispensable in the uterus (Figure 2). It is shown that epithelial PR is required for P_4_−mediated inhibition of uterine epithelial cell proliferation [30]. This discrepancy may be due to differences in treatment design (P_4_ for 3 days and then E_2_+P_4_ for 1 day *vs*. E_2_+P_4_ for 7 days), E_2_ doses (50 ng *vs*. 1 μg), mouse genetic background (B6.SJL.129 mixed *vs*. FVB) and/or housing environment. We note that 7−day treatment with E_2_ and P_4_ might be pharmacologic rather than physiologic. Nonetheless, we believe our results are biologically relevant because P_4_−mediated inhibition of uterine epithelial proliferation during pregnancy depends on Hand2 transcription factor [31]. Expression of Hand2 depends on P_4_ and PR and is restricted in the uterine stroma [31].

P_4_ inhibited E_2_−induced apoptosis in the uterus of both *Pgr^f/f^* and *Pgr^ed/ed^* mice (Figure 4E-4F). It is consistent with that P_4_ prevents epithelial cell apoptosis in the tissue recombinant composed of *Pgr^+/+^* stroma and *Pgr^−/−^* epithelium [16]. E_2_ induces apoptosis in the uterine epithelium of *Esr1^f/f^* and *Esr1^ed/ed^* mice [13]. These results suggest that stromal ERα promotes and stromal PR inhibits apoptosis of uterine epithelial cells. One possibility is that stromal PR activates expression of a paracrine factor(s) that promotes cell survival. PR interacts with ERα and modulates the function of ERα in breast cancer cells [32]. Another possibility is that PR interacts with ERα in uterine stromal cells and inhibits ERα−mediated expression of a pro−apoptotic secretory factor(s). In the rodent uterus, E_2_−induced hyperplasia is eliminated by apoptosis [33]. Perhaps, E_2_−induced apoptosis is to balance out E_2_−induced hyperproliferation of uterine epithelial cells. Consistent with this idea, the apoptotic index was correlated with the proliferative index in the uterine epithelium (Figure 2F and 4F). Unlike the uterus, P_4_ promoted apoptosis in the cervical and vaginal epithelium, which was dependent on epithelial PR (Figure 4A-4D). Epithelial PR was necessary for P_4_ to increase the ratio of nucleus to cytoplasm and suppress expression of K10 in the vagina but not cervix (Table 1). These results reveal that epithelial PR is differentially required for P_4_ responses of epithelial cells in the cervix, vagina, and uterus.

Using mouse genetic models, we determined that ERE binding (i.e., classical mechanism) of ERα is required for upregulation of *Pgr* in the cervix, vagina, and uterus (Figure 5B-5D, 6C, 7 and Supplementary Figure S3). The ERα DBD mutant (E207A/G208A) has acquired the capability to bind hormone response elements such as progesterone response element [29]. We do not believe that the gain−of−function compromises our conclusion because this ERα mutant did not bind to the *Pgr* locus (Figure 7). It is possible that the ChIP−seq analyses might have failed to identify weak ERα−binding sites; thus, we cannot rule out a possibility that the ERα tethering mechanism also contributes to transcription of *Pgr* in the female reproductive tract. Knock−in mice expressing an ERα mutant defective for the tethering mechanism would be required to test this. Reporter assays have shown that AP-1 and Sp-1 sites at the promoter downstream are required for ERα−mediated *PGR* activation in MCF7 breast cancer cells [7, 8]. In the uterus, strong E_2_−induced DBD−dependent ERα binding occurred mostly at far upstream (> 60 kb) of the *Pgr* gene (Figure 7A). Genome−wide enrichment of ERα binding to promoter−distal regions has been identified [28, 34]. ERE−dependent long−range activation of *PGR* by ERα has been demonstrated in MCF7 cells [6]. It is likely that ERα binding to *Pgr* in the uterus occurs in stromal cells because epithelial ERα was not required for upregulation of stromal *Pgr* and downregulation of epithelial *Pgr* (Figure 6B). We postulate that similar long−range regulation by ERα is involved in transcriptional activation of *Pgr* in the cervix and vagina. The AF2 domain was also required for upregulation of *Pgr* by ERα in the cervix, vagina, and uterus (Figure 5B-5D, 6C and Supplementary Figure S3). Upregulation of *Pgr* was evident in the cervical and uterine stroma, but not vagina of *Esr1^neo/neo^* mice (Figure 5C, 6C and Supplementary Figure S3). In tissue recombinants derived from *Esr1^neo/neo^* mice, PR expression is induced by E_2_ in the uterine but not vaginal stroma [5]. These results indicate that (1) ERα DBD and AF2 are commonly required for activation of *Pgr* in the female reproductive tracts and (2) AF1 is required for upregulation of *Pgr* only in the vaginal and cervical epithelium and vaginal stroma. Differential requirement of ERα domains for activation of *Pgr* may be due to distinct chromatin structures in different tissues and cell types, which may require different coactivators [2, 35].

E_2_ suppressed expression of *Pgr* in the uterine epithelium *via* stromal ERα (Figure 6B), which is in agreement with other findings [5, 13]. Downregulation of *Pgr* in the uterine epithelium involves transcriptional repression [5, 27]. It is unlikely that the non−cell autonomous downregulation of *Pgr* is through a cell−cell contact because epithelial and stromal cells are physically separated by the basement membrane. Thus, we postulate that a paracrine factor(s) produced by stromal cells is responsible for repression of *Pgr* in the uterine epithelium. ERα DBD, AF1, and AF2 were required for downregulation of *Pgr* in the uterine epithelium (Figure 6C), further suggesting that ERα DBD, AF1, and AF2 are necessary for regulating expression of such a factor(s). The AP-1 site at the +745 bp region of *PGR* has been implicated in repression of *PGR* in MCF7 cells [9]. It is plausible that a paracrine factor activates AP-1, which in turn suppresses transcription of *Pgr* in the uterine epithelium.

Our results demonstrate distinct functions of epithelial PR in the cervix, vagina, and uterus, suggesting that PR regulates unique sets of genes and pathways in these tissues. Warranted are further studies to identify PR target genes and pathways in the stroma and epithelium of these tissues. Our results also demonstrate unique mechanisms of *Pgr* regulation in the cervix, vagina, and uterus. Together, our results underscore the complexity of function and regulation of *Pgr* in the female reproductive tracts.

## MATERIALS AND METHODS

### Ethics statement

Investigation has been conducted in accordance with the ethical standards and according to national and international guidelines. All procedures were carried out according to animal protocols approved by the University of Houston Institutional Animal Care and Use Committee.

### Mice and treatments

All *Esr1* and *Pgr* mice as well as *Esr2* knockout and *Wnt7a-Cre* transgenic mice were described previously [13, 22–24, 36–39], and genotyped by PCR. All mouse strains except for *Esr2* knockout mice (C57BL/6) were backcrossed to FVB at least 5 generations. Mice were ovariectomized at the age of 6-8 weeks and rested for 2 weeks. They were then i.p. injected with vehicle, 17β-estradiol (E_2_) alone, or E_2_ (1 μg/day) plus P_4_ (1 mg/day) for 7 days.

### Histological staining

The female reproductive tracts were harvested 24 hours after final hormone injections and processed as described previously [19]. Hematoxylin & eosin (H&E) and Alcian blue staining were carried out as described previously [19].

### Immunohistochemistry and immunoblot

Antibodies were purchased from Santa Cruz Biotechnology [ERα (H184), PR (H190) and GAPDH (V-18)], Thermo Scientific [Ki67 (clone SP6) and K10 (clone DE-K10)], BioLegend (K14), GenDEPOT (HRP−conjugated anti−rabbit/goat IgG), Life Technologies (Alexa488−conjugated anti−rabbit/mouse IgG) and Rockland Immunochemicals (biotinylated anti−rabbit IgG). For IHC, sections were deparaffinized, rehydrated, microwaved in 10 mM sodium citrate buffer (pH 6.0) and incubated with primary antibodies diluted in blocking buffers (PR, 1:200 in 5% goat serum; ERα, 1:100 in 10% goat serum and 0.5% skim milk; Ki67, 1:100 in 10% goat serum; K10, 1:50 in 5% goat serum). After extensive washes, sections were incubated with Alexa488−conjugated anti−rabbit/mouse IgG or biotinylated anti−rabbit IgG followed by ABC complex (Vector Laboratories) as described previously [19]. For immunoblot, cervical tissue homogenates were resolved in 7.5% SDS−polyacrylamide gel and proteins were transferred to PVDF membranes.

### TUNEL assay

TUNEL staining was carried out using ApopTag Fluorescein *in situ* apoptosis detection kit (Millipore) according to the manufacturer's instructions.

### Microscopy and digital image analyses

Stained tissue sections were visualized by an Olympus BX51 or a Nikon ECLIPSE Ti microscope, and photographed with a color camera (Olympus DP73) or cooled CCD monochrome cameras (Olympus XM10 and Photometrics CoolSNAP HQ2). Digital images were acquired with imaging software (Olympus cellSens Dimension and Nikon NIS-Elements AR). For quantification, images were acquired from 5 microscopic fields per tissue with a 20X objective lens, and cells were quantified using the counting function of the NIH ImageJ software. Typical ranges of total cell count per tissue were 400 - 600 in the basal layer and 1,500 - 2,000 in the suprabasal layers.

### Chromatin immunoprecipitation and quantitative real−time PCR

Chromatin was prepared from ovariectomized *Esr1^+/+^* or *Esr1^AA/−^* uterus one hour after i.p. injection with saline or E_2_ (250 ng/mouse) and ERα was immunoprecipitated as described previously [29]. Candidate ERα−binding regions in the *Pgr* locus were amplified using qPCR as described previously [29]. Nanograms of amplimer DNA was calculated using a standard curve, and normalized to % input DNA for each sample. Primers for the −62 kb region were 5′-GGGTCGAAACTACCAGCTAAA-3′ (forward) and 5′-CAAAGGCTTGGACAAATGAA-3′ (reverse). Primers for intron 3-4 were 5′-TCTGCTCCAATGACTGTGTTC-3′ (forward) and 5′-ATCACATGCACTGAGAAGATCA-3′ (reverse). To compare expression levels of *Pgr* mRNA in the cervix, qPCR was carried out using a SYBR Green detection method. The relative mRNA levels were calculated using the ΔΔC_t_ method with *Ppia* mRNA as control. Primers for *Pgr* were 5′-CCAGCATGTCGTCTGAGAAA-3′ (forward) and 5′-GCCTGGCTCTCGTTAGGAAG-3′ (reverse). Primers for *Ppia* were 5′-GGGTTCCTCCTTTCACAGAA-3′ (forward) and 5′-GATGCCAGGACCTGTATGCT-3′ (reverse).

### Statistical analyses

Wilcoxon rank sum test was used for analysis of cell proliferation and apoptosis. Student's *t*-test or 2-way ANOVA with Fisher's LSD was used for analysis of qPCR data.

## SUPPLEMENTARY MATERIAL FIGURES


