# Supplementary material for: Distinct functions and regulation of epithelial progesterone receptor in the mouse cervix, vagina, and uterus
Source: Oncotarget. 2016 Mar 17;7(14):17455–67. doi: 10.18632/oncotarget.8159 (PMC4951225; doi:10.18632/oncotarget.8159)
Supplement: Supplementary file 1 [file oncotarget-07-17455-s001.pdf]

Distinct functions and regulation of epithelial progesterone receptor in the mouse cervix, vagina, and uterus

Supplementary Material

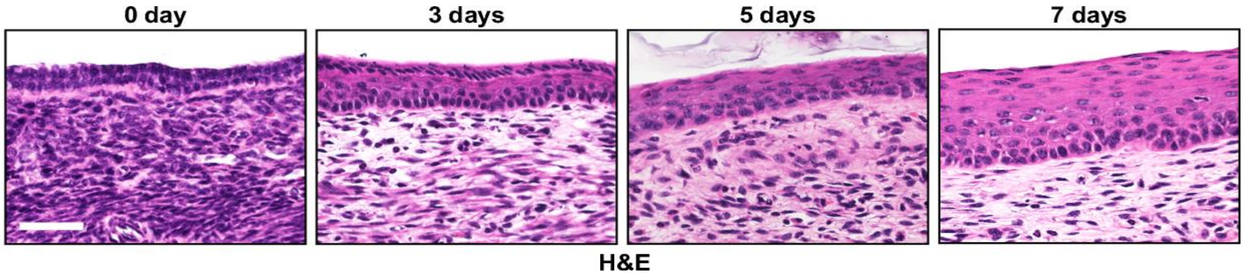

Supplementary Figure S1: E_2 treatment for 7 days is required for the cervical epithelium to reach the full thickness. Ovariectomized mice were treated with E_2 for indicated durations. All mice were sacrificed 3 weeks after ovariectomy. Shown are representative images of the cervix stained with H&E (n=3). Scale bar, 50 µm.

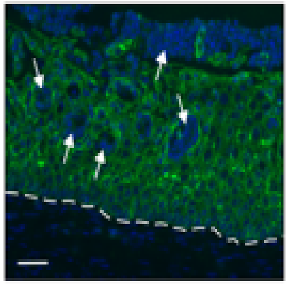

Supplementary Figure S2: Cells infiltrating the vaginal epithelium upon P_4 treatment are negative for K14. Ovariectomized *Pgr*$^{f/f}$ mice were treated with E_2+P_4 for 7 days. Vaginal sections were stained for K14 (green). Nuclei were stained with Hoechst 33342 (blue). The dotted line separates epithelium and stroma. Arrows point to K14–negative cells. Note that vaginal epithelial cells should express K14. Scale bar, 30 μm.

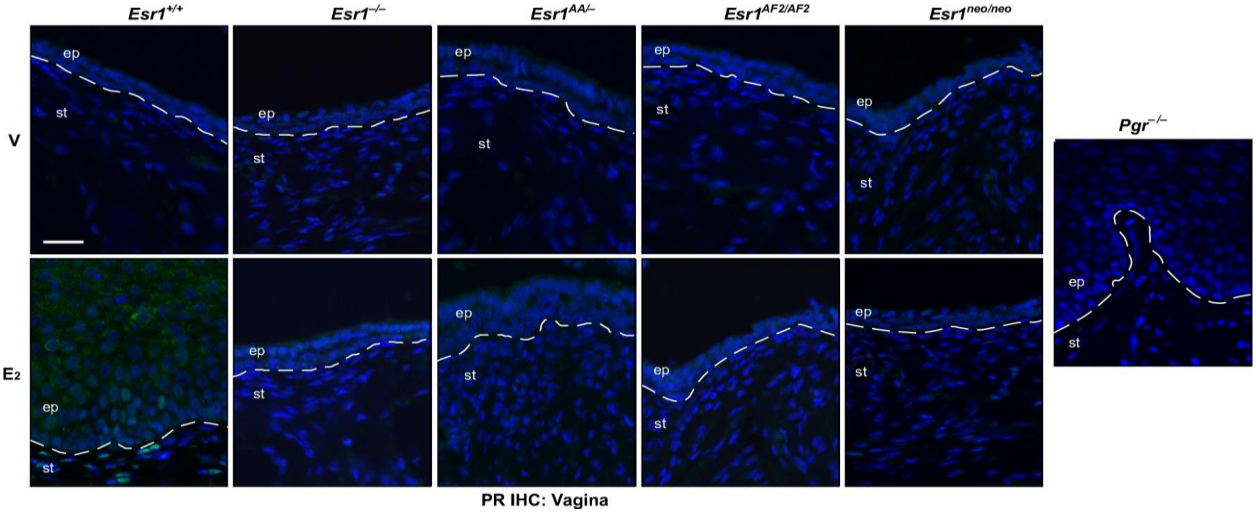

Supplementary Figure S3: ERα DBD, AF1, and AF2 are required for upregulation of *Pgr* in the vagina. *Esr1* mutant mice were ovariectomized and treated with vehicle (V) or E_2. Vaginal sections were stained for PR (green). Nuclei were stained with Hoechst 33342 (blue). Dotted lines separate epithelium (ep) and stroma (st). Scale bar, 25 μm.

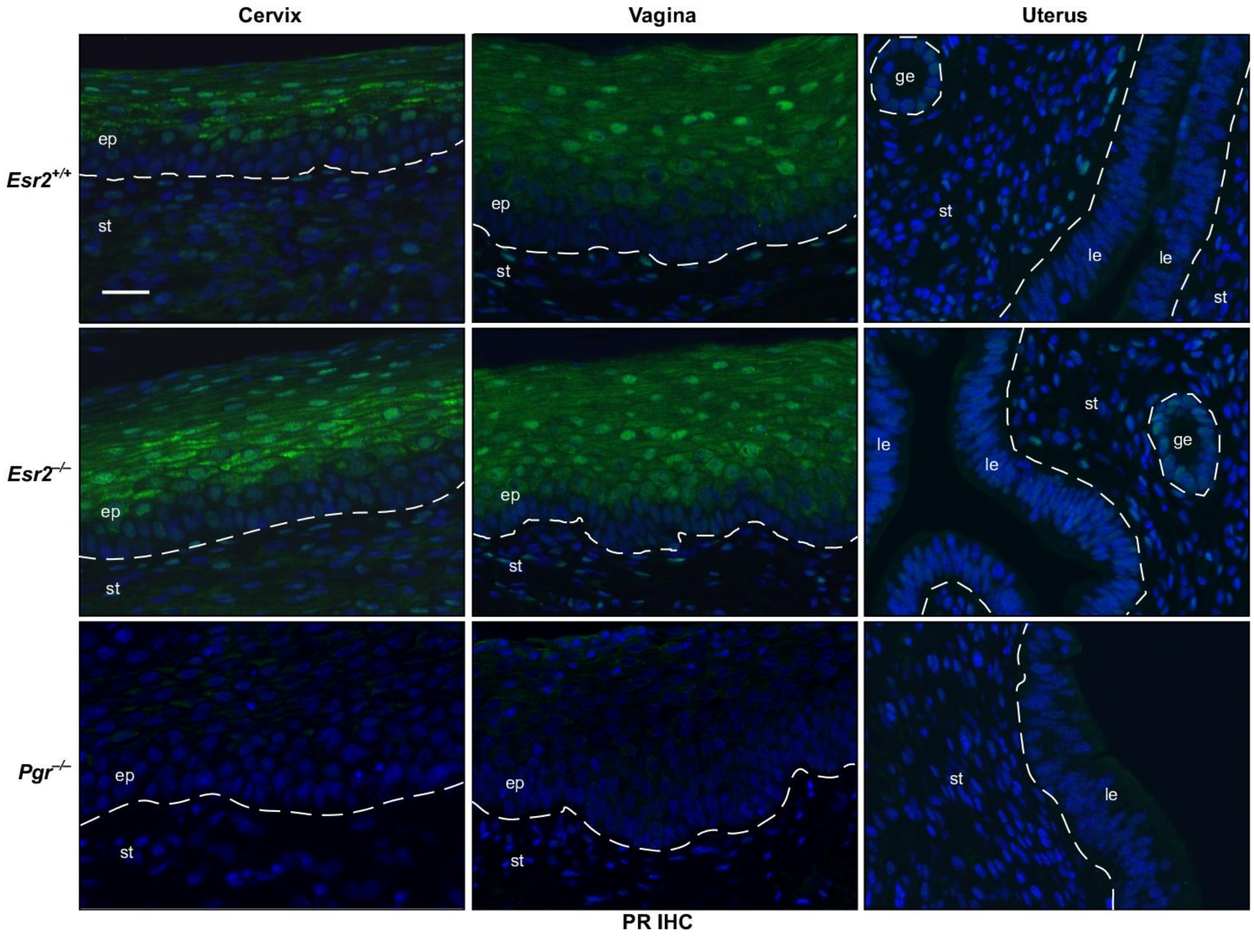

Supplementary Figure S4: ERβ is not required for E_2–mediated regulation of *Pgr* in the female reproductive tract. *Esr2*$^{-/-}$ mice were ovariectomized and treated with E_2. Cervical, vaginal, and uterine sections were stained for PR (green). Nuclei were stained with Hoechst 33342 (blue). Note that PR staining patterns in *Esr2*$^{+/+}$ and *Esr2*$^{-/-}$ mice were similar. Dotted lines separate stroma (st) from cervical/vaginal epithelium (ep) and uterine luminal (le)/glandular epithelium (ge). Scale bar, 25 μm.
